# Exploring the Roots of Diabetes: Bisphenol A May Promote Insulin Resistance

**Published:** 2006-01

**Authors:** Cynthia Washam

Poor diet and lack of exercise are known contributors to the epidemic of type 2 diabetes spreading around the world. Now researchers have implicated another possible culprit in the rise of the disease **[*EHP* 114:106–112]**. A team of Spanish and Mexican researchers reports discovering that the endocrine-disrupting chemical bisphenol A (BPA) causes insulin resistance in mice similar to that seen just before the onset of type 2 diabetes.

Type 2 diabetes occurs when insulin receptors throughout the body fail; this is known as insulin resistance. Complications of diabetes include heart disease, kidney failure, blindness, and nerve damage. The World Health Organization estimates that at least 154 million people around the world have type 2 diabetes, and predicts that number will more than double within 25 years.

Endocrine disruptors mimic the natural sex hormone 17β-estradiol (E_2_), which is involved in the development of sexual traits. Scientists have known for years that BPA and other endocrine disruptors can diminish sperm production, accelerate the onset of puberty, and damage sexual organs. But they had not studied a link between the chemicals and glucose metabolism, even though increases in E_2_ are known to cause insulin resistance.

The researchers chose to study BPA because its use is so widespread. Since the 1950s, it has been used in plastics for water bottles and jugs, baby bottles, toys, and the linings of food and beverage cans. People ingest BPA that leaches from containers into foods and drinks. Studies in the United States showed that BPA appeared in the blood and urine of 95% of people tested.

The researchers tested BPA’s effect on glucose regulation by measuring glucose and insulin levels in adult male mice treated with BPA injections, then comparing them with levels in mice treated with E_2_ and a control group treated with corn oil. BPA caused oversecretion of insulin in mice at a dose of 10 micrograms per kilogram body weight per day (μg/kg/day) via a rapid mechanism, taking only 15 to 30 minutes. Treatment over a course of four days with 100 μg/kg/day induced the insulin resistance that precedes type 2 diabetes. E_2_ had the same effects at the same doses. Glucose metabolism remained stable in the control rats.

These results are novel because the mechanism reported is the lesser known of the two major pathways used by estrogens and other steroids. It involves signaling rapidly initiated from the plasma membrane rather than the nuclear transcription pathway depicted in most textbooks.

The BPA dose high enough to cause insulin resistance in mice was in the same range as the 50 μg/kg/day reference dose established by the U.S. Environmental Protection Agency, which is based on a lowest-observed-adverse-effect level of 50 milligrams per kilogram per day. The researchers see the newly discovered link between BPA and insulin resistance as one more reason the agency should at least consider lowering the lowest-observed-adverse-effect level. They further suspect that because other endocrine disruptors mimic E_2_, they too may hinder glucose metabolism.

